# Genetic and environmental influences on circulating NK and T cells and their relation to bipolar disorder

**DOI:** 10.1186/s40345-018-0139-3

**Published:** 2019-02-10

**Authors:** G. Snijders, R. Brouwer, S. Kemner, F. Bootsman, H. A. Drexhage, M. H. J. Hillegers

**Affiliations:** 1 Department of Psychiatry, Brain Center Rudolf Magnus, University Medical Center Utrecht, Heidelberglaan 100, P.O. Box 85500, 3508 GA Utrecht, The Netherlands; 2 Department of Immunology, Erasmus Medical Center, Rotterdam, The Netherlands; 3 Department of Child and Adolescent Psychiatry/Psychology, Erasmus Medical Center-Sophia Children’s Hospital, Rotterdam, The Netherlands

**Keywords:** Bipolar, T cells, NK cells, Environment, Genes

## Abstract

**Background:**

In previous studies we found mild deficiencies of circulating T cells in patients with bipolar disorder (BD) and children at risk for BD, correlating to a higher inflammatory state. The genetic and environmental influences on these T cell deficiencies in association with BD development are unknown.

**Objectives:**

The aim is to quantify genetic and environmental factors that contribute to the association between the liability to develop BD and T cell deficiencies.

**Methods:**

Participants of a Dutch bipolar twin study (11 monozygotic BD twin pairs, 15 dizygotic BD twin pairs, 15 monozygotic and 12 dizygotic healthy twin pairs) were included. A detailed FACS analysis of frozen stored leukocytes was carried out to determine the percentages of T cells and various other leukocyte and lymphocyte subsets. A bivariate liability threshold twin model was used to determine genetic and environmental (common and unique) influences on the correlation between BD and the various subsets.

**Results:**

Lower percentages of T cells and higher percentages of NK cells were associated with the familial liability to develop BD. Neither genetic nor shared or unique environmental factors could explain the associations. Lithium usage explained part of the association for T cells, smoking in part that for NK cells.

**Conclusions:**

Our results confirm that BD is the result of a complex interaction between various genetic and environmental risk factors, in which T and NK cells act as important intermediate immune players.

## Introduction

The origin of bipolar disorder (BD) has a multitude of factors, both genetic influences and environmental factors play a role in the pathogenesis. Chronic low grade inflammation is presently thought to play an important role as one of such factors, in at least part of the patients. In a previous twin study we showed that a, not yet identified, common environmental factor, shared between twins, dominates the association between BD and the raised level of inflammation-related genes in circulating monocytes (Padmos et al. 2009).

We also gathered evidence that T cell abnormalities play a role in the development of the chronic low grade inflammatory state of BD. We detected in four separate cohort studies that the T cell system is set at another equilibrium in BD and we found mild deficiencies in the number of CD^3+^ T cells compared to such values in healthy controls (HC) (Drexhage et al. 2011; Snijders et al. 2016; Bergink 2014; Vogels et al. 2017). Within the mildly deficient CD^3+^ T cell population there were abnormal fluctuations over time in the circulating levels of CD^4+^ T regulatory (Treg), T helper 2 (Th2), T helper 1 (Th1) and T helper 17 (Th17) cells. (Drexhage et al. 2011; Snijders et al. 2016; Bergink 2014; Vogels et al. 2017). Mild deficiencies in the percentages of Treg cells correlated to raised levels of inflammation-related genes in circulating monocytes (Snijders et al. 2016). Breunis et al. (2003) reported higher percentages of activated CD^3+^ T cells within overall reduced percentages of CD^3+^ T cells in BD patients as compared to HC, particularly in manic patients (Breunis et al. 2003). Wieck et al. (2013) and Barbosa et al. (2014) reported reduced percentages of Treg cells emerging against a background of abnormal circulating levels of Th1, Th2 and Th17 cells in BD patients when compared to HC (Wieck et al. 2013; Barbosa et al. 2014).

All together, these findings suggest the existence of a mild deficiency of CD^3+^ T cells in at least part of BD patients with fluctuating numbers of pro-and anti-inflammatory T cell subsets over the course of the disease, influencing a state of low grade inflammation in these patients.

Twin studies are suitable to differentiate whether these mild T cell deficiencies are due to genetic or (shared/unique) environmental factors. Therefore, we quantified-using FACS analysis and a bivariate liability threshold twin model-common and unique environmental contributions to the association between BD liability and circulating levels of T cells and T helper cell subsets. Using FACS we were also able to investigate the effect of other circulating leukocyte subsets playing a role in cell-mediated immunity and described to be abnormal in BD, such as the monocytes and natural killer (NK) cells.

## Methods

### Patients and procedures

The described (bipolar) twins herein belong to the longitudinal Dutch bipolar twin study of the University Medical Center Utrecht (UMCU). Study design, recruitment procedure and study population have been described in detail elsewhere (Vonk et al. 2007; van der Schot et al. 2009). Numbers and details on the included twins are given in Table 1. Addressing the aim of this study, additional analysis has been performed regarding mood state. Current mood states were assessed using the Young Mania Rating Scale (YMRS) (Young et al. 1978) and the Inventory for Depressive Symptomatology (IDS) (Rusha et al. 1996). At time of the study, 11 patients were in a mood episode, defined as YMRS > 13 and/or IDS > 7, and 17 were euthymic, defined as YMRS ≤ 13 and/or IDS ≤ 7. 46% of cases had missing values for current mood state, these cases were not included in the analysis. This study received approval by the Medical Ethics Committee of UMCU. Written informed consent was obtained from all participants after a complete description of the study was given.

**Table 1:** Demographic characteristics

	Bipolar twins (BD)					Control twins (HC)		
	Total (n = 52)	MZ (n = 22)	DZ (n = 30)	Index (n = 26)	Co-twin (n = 26)	Total (n = 54)	MZ (n = 30)	DZ (n = 24)
Age, mean (SD), range, y	49.6 (10.2), 29–69	47.5 (11.0), 29–62	51.1 (9.5), 37–69	49.6 (10.2), 29–69	47.5, (11.0), 29–62	48.3 (8.4), 32–72	48.5 (10.2), 32–72	48.1 (6.0), 39–57
Female, n (%)	37 (71.2)	18 (81.8)	19 (63.3)	19 (73.1)	18 (69.2)	38 (70.3)	24 (80.0)	14 (58.3)
Diagnosis								
Bipolar disorder I	20	7	13	20	–	–	–	–
Bipolar disorder II	9	6	3	6	3	–	–	–
Bipolar Disorder	1	–	1	–	1	–	–	–
NOS								
Unipolar disorder	4	3	1	–	4	2	0	2
Psychotic disorder	3	1	2	–	3	–	–	–
Other disorder	4	1	3	–	4	8	4	4
No disorder	11	4	7	–	11	45	27	18
Mood								
Euthymic	17	8	9	14	3			
Mood episode	11	5	6	10	1			
Missing data	24	9	15	2	22			
Age at onset, mean (SD), range, y	26.9 (10.3), 13–57	24.4 (7.8), 13–40	29.4 (11.9), 14–57	29.6 (10.7), 16–57	22.3 (7.5), 13–40			
Smoking, n (%)	28 (53.8)	10 (45.5)	18 (60.0)	17 (65.4)	11 (42.3)	27 (50.0)	15 (50.0)	12 (50.0)
Body mass index, mean (SD) range	25.1 (4.4), 17–42	25.5 (4.7), 18–42	24.9 (4.1), 17–28	26.1 (5.0), 17–42	24.2 (3.4), 18–31	25.2 (4.7), 18–40	24.4 (3.4), 19–30	26.2 (5.9), 18–40
Psychotropic medication								
Lithium	16	7	9	14	2	0	0	0
Anticonvulsants	3	1	2	2	1	0	0	0
Antipsychotics	18	5	13	13	5	0	0	0
Antidepressants	10	4	6	7	3	1	1	0
Benzodiazepines	8	4	4	7	1	1	1	0

*BD* bipolar disorder, *HC* healthy controls, *DZ* dizygotic, *MZ* monozygotic

Mood episode, defined as YMRS > 13 and/or IDS > 7. Euthymic, defined as YMRS ≤ 13 and/or IDS ≤ 7. Current use of psychotropic medication is reported. No significant differences between different groups, except for use of psychotropic medication (*p* < 0.001)

Blood collection, leukocyte preparation and the Fluorescence Activating Cell Sorting (FACS) analysis have been described in detail before (Bergink 2014; Snijders et al. 2016; Vogels et al. 2017).

### Statistical analysis

Genetic modelling was carried out using the OpenMx package (Kenny et al. 2009) in R statistical software. A bivariate liability threshold model was used to model the data. The variance of a certain trait (such as lymphoid subsets) or the covariance between two traits (lymphoid subset and the liability to develop BD) can be due to additive genetic (A); common environment (C), and unique environmental factors (E). Twin models focus on the difference in resemblance for a trait between monozygotic (MZ) twins who share (nearly) 100% of their genes relative to dizygotic (DZ) twin pairs who share on average 50% of their segregating genes. This means that the additive genetic effects acting on members of MZ pairs correlate fully (correlation 1), whereas DZ correlation is 0.5. For both MZ and DZ twins, shared environment factors are fully correlated by definition. The model does not assume correlations between unique environment factors and these are set to 0. A larger correlation between trait(s) in MZ twins than DZ twins indicates that genetic factors play a role, but if the MZ correlation is smaller than twice the correlation in DZ twins, then shared environmental factor are thought to play a role.

To estimate the magnitude effects of A, C and E on the different lymphoid subsets a bivariate twin model was used. The relative contribution of A, C and E to the variance of lymphoid subsets was expressed as the percentage of genetic/common environment and unique environment in total variance. The univariate heritability is calculated with the equation; h^2^ = A/(A + C + E). Common environmental influences (c^2^) and unique environmental influences (e^2^) are defined similarly. Disease status was dichotomous and assumed to represent an underlying continuous liability with a mean of 0 (SD = 1). A bipolar patient will have a high value on the liability scale, thereby crossing a certain threshold (patient status = 1). All discordant co-twins or controls will have lower liability scores and will not cross the critical threshold (patient status = 0). The bivariate liability threshold twin model elucidate genetic, common environmental and unique environmental contributions to the association between lymphoid subsets and BD liability by investigating cross-trait cross-twin correlations. We could not estimate heritability or prevalence from this sample, since twin pairs in our sample were specifically selected for BD. Therefore, prevalence and heritability of BD were fixed to population values (1% prevalence, h^2^ = 70%, c^2^ = 15% and, e^2^ = 15%) (Regeer et al. 2004). Percentages of Th1, Th2, Th17 and Tregs were non-normally distributed; therefore the log transformation (log_10_) was used prior to analysis. As post hoc analyses we repeated the genetic modeling including age and sex as covariates. Current lithium use, BMI and smoking status were separately included as additional covariates (together with age and sex) in bivariate twin models. A multivariate analysis of variance (MANOVA) was performed to test for overall significant differences between different subgroups. Significant MANOVAs were post hoc followed by a univariate analysis of variance (ANOVA). Outliers, defined as 3 standard deviations ≤ or ≥ the mean level) were excluded for further analysis.

## Results

Significant socio-demographic and clinical differences between MZ/DZ and BD/HC twins were not found, except for the use of psychotropic medication between BD patients and HC (Table 1).

### Effect of genes and environmental factors on lymphocyte subpopulations

Heritability of the investigated leukocyte subpopulations was estimated (Table 2A). The circulating levels of Th17 and T regulatory cells were predominantly explained by genes (h^2^: 79 and 69%, respectively), the remaining part was largely explained by unique environmental factors (e^2^: 21 and 28%, respectively).

**Table 2:** Effect of genes and environmental factors on lymphoid subsets in relation of bipolar disorder

Cell population	A. Sources on variance on lymphoid subsets, irrespective of disease (n = 106), % (95% CI)			Phenotypic correlation	
	h^2^	c^2^	e^2^	Rph	p
T helper 17 cells	0.79 (0.35–0.88)	0.009 (NA–0.37)	0.21 (0.11–0.38)	0.02 (− 0.17–0.22)	0.80
T regulator cells	0.69 (0.17–0.85)	0.029 (NA–0.39)	0.28 (0.14–0.55)	0.06 (− 0.13–0.25)	0.53
Total T helper cells	0.59 (0.04–0.87)	0.18 (0.00–0.63)	0.22 (0.12–0.42)	− 0.06 (−0.25–0.13)	0.54
Total T cells	0.57 (0.00–0.82)	0.11 (0.00–0.57)	0.32 (0.18–0.59)	− 0.21 (− 0.38–0.02)	0.03*
T helper 2 cells	0.51 (0.01–0.86)	0.27 (0.00–0.67)	0.22 (0.11–0.47)	0.10 (− 0.09–0.28)	0.30
NK cells	0.50 (0.00–0.82)	0.21 (0.00–0.62)	0.28 (0.14–0.54)	0.18 (0.00–0.34)	0.04*
Monocytes	0.25 (0.09–0.43)	0.30 (0.11–0.51)	0.45 (0.28–0.69)	0.15 (− 0.03–0.32)	0.10
T helper 1 cells	0.19 (0.00–0.74)	0.38 (0.00–0.69)	0.43 (0.24-0.69)	− 0.05 (− 0.23–0.13)	0.57

Cell population	B. Bipolar twins and their co-twins (n = 52)	Healthy control twins (n = 54)				
	M (S.E.)^a^	M (S.E)^a^	P (MANCOVA/ANCOVA^b^)			
T helper 17 cells	0.29 (1.07)	0.27 (1.07)	0.349			
T regulator cells	1.92 (1.04)	1.83 (1.04)	0.698			
Total T helper cells	38.05 (1.18)	38.37 (1.18)	0.402			
Total T cells	54.48 (1.18)	59.0 (1.17)	0.015/0.007*			
T helper 2 cells	0.68 (1.06)	0.62 (1.6)	0.457			
NK cells	12.62 (0.66)	10.01 (0.63)	0.009/0.006*			
Monocytes	18.53 (0.81)	16.86 (0.81)	0.181			
T helper 1 cells	5.47 (1.07)	5.97 (1.06)	0.553			

The different lymphoid subsets and NK cells are displayed in order of highest to lowest source of genetic variance

T helper 17 cells: CD^4+^ IL17A. T regulatory cells: CD^3+^ CD25 highFoxP3+. Total T helper cells: CD^3+^ CD^4+^ CD^8−^;Total T-cells: CD^3+^; T helper 2 cells: CD^4+^ IL-4+; NK cells: CD^3+^–CD^56+^; Monocytes: CD^14+^, T helper 1 cells: CD4+ IFN-y+

Monocytes, T cells, T helper cells and NK cells were expressed in the calculations as percentages of intact CD^45+^ PBMC

The subsets of T helper cells (T helper 17, T helper 2, T helper 1 and T regulator cells) were expressed in the calculations as percentages of lymphocytes

* Significant at p = 0.05, uncorrected for multiple comparisons

^a^To normalize data some values were transformed, for interpretation the means and standard errors were back transformed

^b^*p* value based on multivariate analysis (MANOVA). A follow-up univariate analysis with post hoc least significance difference (LSD) analysis was performed for NK cells and total T cells

Half of the variance of the circulating levels of T helper 2 cells and NK cells was determined by genetic factors (h^2^: 51% and 50%, respectively), whilst unique and common environmental factors equally contributed to the remaining part of the total variance.

The proportion of total variance in circulating numbers of monocytes and T helper 1 cells was mainly determined by unique and common environmental factors and only for a small part (a quarter) by genetic factors.

### Association between the liability to develop bipolar disorder and the circulating percentages of T cells and NK cells

As shown in Table 2A, of the leukocyte populations determined the circulating populations of T cells and NK cells showed significant correlations with the phenotype BD. These were thus further evaluated.

#### T cells

A significant negative phenotypic association between the liability to develop BD and the percentage of T cells was found (*r*_*ph*_= − 0.21, *p *= 0.03). Post-hoc analysis showed significantly lower mean levels of percentages of T cells in the index and their co-twins as compared to the HC twins (*p *= 0.007) (Table 2B). T cell percentages were not significantly different in the group of BD patients with an active mood episode versus those in euthymic state (50.5% resp. 55.9%, r_*s*_ = − 0.31, *p* = 0.12). The covariance of the number of T cells with BD could not be attributed to predominant genetic or common environmental contributors. Including age and sex as covariates did not alter the results (data not shown).

Together with age and sex we corrected for lithium treatment, BMI and smoking separately. The phenotypic association between the liability to develop BD and the circulating levels of T cells lost significance after including lithium treatment as confounding factor in the bivariate liability threshold model (r_*ph*_ = − 0.15 *p *= 0.107), indicating a role for lithium in this negative association. However, also the co-twins of the BD index twin without any psychiatric disorder or the co-twins of the BD index twin with another psychiatric disorder (both not using lithium) showed reduced percentages of T cells as compared to the healthy twins, be it only statistically significant for the co-twins with another psychiatric disorder (52.9 (2.60), respectively 59.0 (1.17), *p* = 0.034).

#### NK cells

The phenotypic association between the liability to develop BD and the circulating levels of NK cells also reached significance (*r*_*ph*_= 0.18, *p *= 0.04). Post-hoc analysis showed significantly higher mean levels of percentages of NK cells in the index and co-twins as compared to the HC twins (*p *= 0.006) (Table 2B). As in the case of the percentage of T cells we could not disentangle the covariance between the liability to develop BD and levels of circulating NK cells in genetic or environmental covariance. Interestingly enough, in the healthy co-twins of the BD index twins significantly higher circulating levels of NK cells were found, indicating an intrinsic familial liability for higher levels of NK cells irrespective of a mood disturbance (data not shown).

Addition of age and sex as covariates did not influence the results. However, the association between circulating levels of NK cells and the liability to develop BD lost some significance after including smoking as an additional confounding factor (together with age and gender) in the bivariate liability threshold model (*r*_*ph*_= 0.18, *p *= 0.06). Correction for BMI and lithium did not affect the outcome.

## Discussion

By a bivariate twin analysis, we showed that reduced levels of circulating T cells are associated with the liability to develop bipolar disorder. Although lithium use was associated with reduced circulating levels of T cells in bipolar index cases, we also found that circulating levels of T cells were reduced in co-twins without BD not taking lithium. Neither genetic nor shared or unique environmental factors explained the association between the decreased circulating levels of T cells and the liability to develop BD. This indicates that probably different and perhaps varying genetic and environmental factors together determine the mild reduction in T cells in the families of the twins liable to BD.

Mild deficiencies of T cells per se could explain disturbances in mood state, since T cells are indispensable for a proper structure and function of the hippocampus (Lewitus et al. 2008; Niebling 2004). Mild to severe T cell deficiencies can be due to genetic defects/polymorphisms, such as to the 22q11 deletion syndrome (Vergaelen et al. 2018). On the other hand environmental influences, such as virus infections (e.g. HIV and measles), and nutritional deficiencies (e.g. protein) may lead to T cell deficiencies. Also stress, related to different states in BD, is capable of triggering significant production of inflammatory cytokines (Glaser et al. 2005), which are capable of inducing an accelerated T cell apoptosis. Recent findings indeed highlight that BD related heritable genetic factors may act in concert with sustained elevated immune-inflammatory signaling pathways (Mühleisen et al. 2014).

This study also shows that higher levels of circulating NK cells were associated with the liability to develop BD. Again neither genetic nor shared or unique environmental factors significantly explained the association. NK cells are considered to be innate immune cells important in combating virally infected cells. Raised numbers of NK cells would in such view be a sign of a virus infection. However, NK cells are also capable of dampening down the activation of microglial cells (Shi et al. 2011), and can thus be viewed as a sort of brain-specific “suppressor cells” for pro-inflammatory activated microglial cells found in the hippocampus of BD patients (Haarman et al. 2015). Moreover, raised numbers of NK cells would be capable of suppressing e.g. inflammatory activated microglia in the brain.

Our report stands out from other reports, since the majority of the reports show normal levels of NK cells in BD (Karpiński et al. 2016), including two of our previous reports (be it that percentages were at the higher end of the normal spectrum (Drexhage et al. 2011; Snijders et al. 2016). It must be noted that, the herein found higher circulating level of NK cells just reached statistical significance (p = 0.04), and hovered at the brink of significance (*p *= 0.06) after correction for smoking. Thus, in sum, the increase in peripheral NK cells does not seem to be a strong characteristic of BD.

### Limitations

Although our study is one of the largest bipolar twin studies, findings are preliminary, and the sample size is still too small for a division in relevant subgroups (duration of disease, somatic comorbidities, such as the metabolic syndrome and cardiovascular disease, or medication use) without losing statistical power. This probably explains why we could not disentangle genetic and environmental influences causing the association between NK, T cells and disease. This not only applies to the aforementioned subdivisions, but also to other confounding factors influencing immune functions. Several lifestyle (e.g. alcohol, obesogenic diet) and environmental factors are known to have effects on cell mediated immunity (amongst which is also smoking, which marginally influenced the outcomes for NK cells). We also were not sufficiently informed about the physical activity and hormonal status (e.g. menstrual cycle) of the twins to be able to correct for that.

Furthermore we have not determined the total number of circulating leukocytes per quantity of blood. We have therefore worked with relative percentages, e.g. the percentage of T cells of the leukocytes in the collected PBMC preparation. It is more appropriate to work with absolute numbers of subsets of leukocytes per quantity of blood in future studies.

In summary and despite these limitations, our results show that reduced percentages of circulating T cells and high percentages of circulating NK cells are linked to the liability to develop BD. However confirmation in larger cohorts, delivering more detail, is essential. Our results make it plausible that the mentioned immune abnormalities are the result of a complex interplay between multiple genetic and environmental risk factors.
